# Supplementary material for: Dietary Walnuts Prevented Indomethacin-Induced Gastric Damage via AP-1 Transcribed 15-PGDH, Nrf2-Mediated HO-1, and n-3 PUFA-Derived Resolvin E1
Source: Int J Mol Sci. 2024 Jun 30;25(13):7239. doi: 10.3390/ijms25137239 (PMC11242660; doi:10.3390/ijms25137239)
Supplement: Supplementary file 1 [file ijms-25-07239-s001.zip › ijms-3049024-supplementary.pdf]

(a)

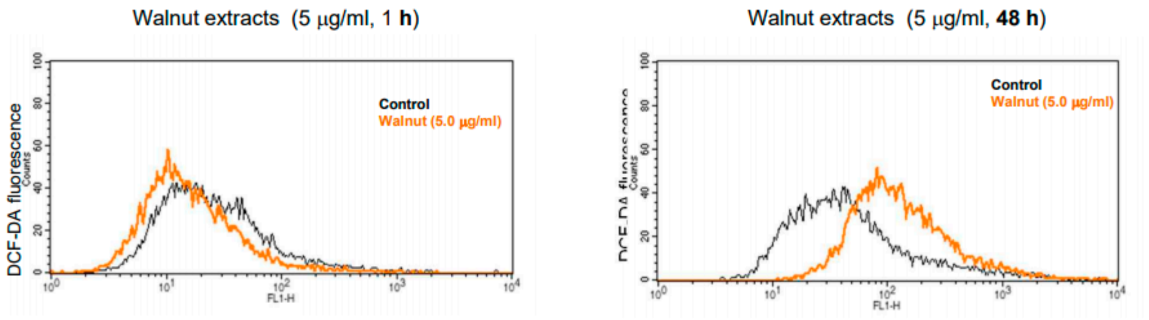

(b)

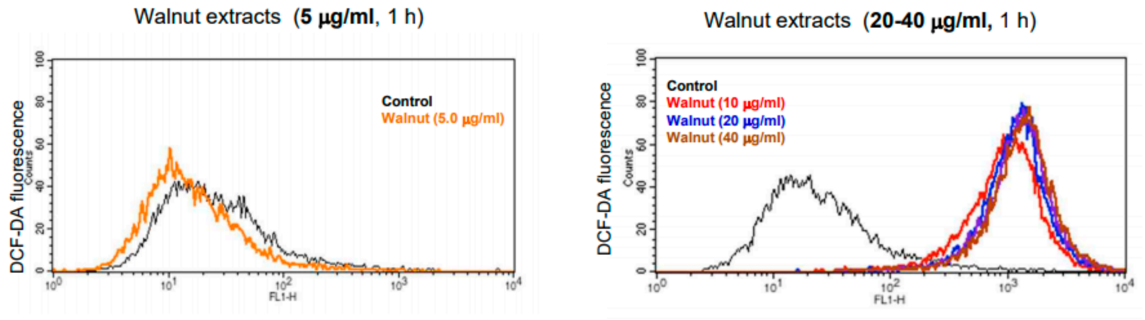

(c)

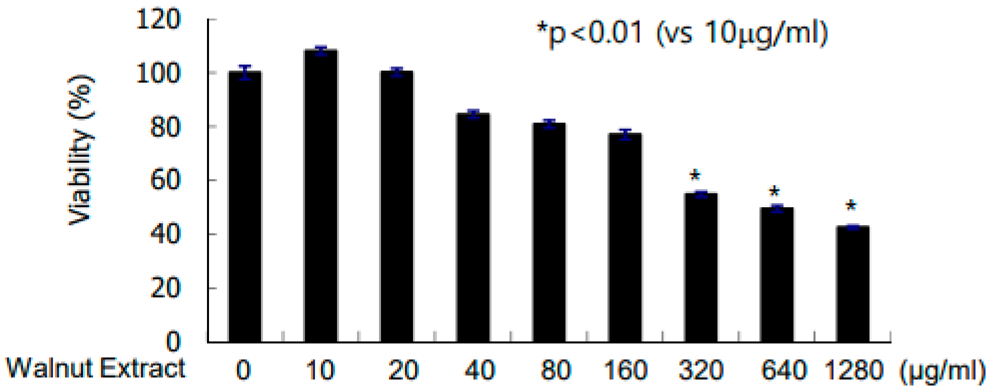

Supplementary Figure S1. DCFDA flow cytometry changes after different times of WPE exposure (a) 5 g/ml WPE for 1hr and 5 g/ml WPE for 48hr (b) 5 g/ml WPE for 1hr and 20-40 g/ml WPE for 1hr (c) Cell viability assay after exposure to different dosing of WPE, 10, 20, 40, 80, 160, 640, and 1280 g/ml.

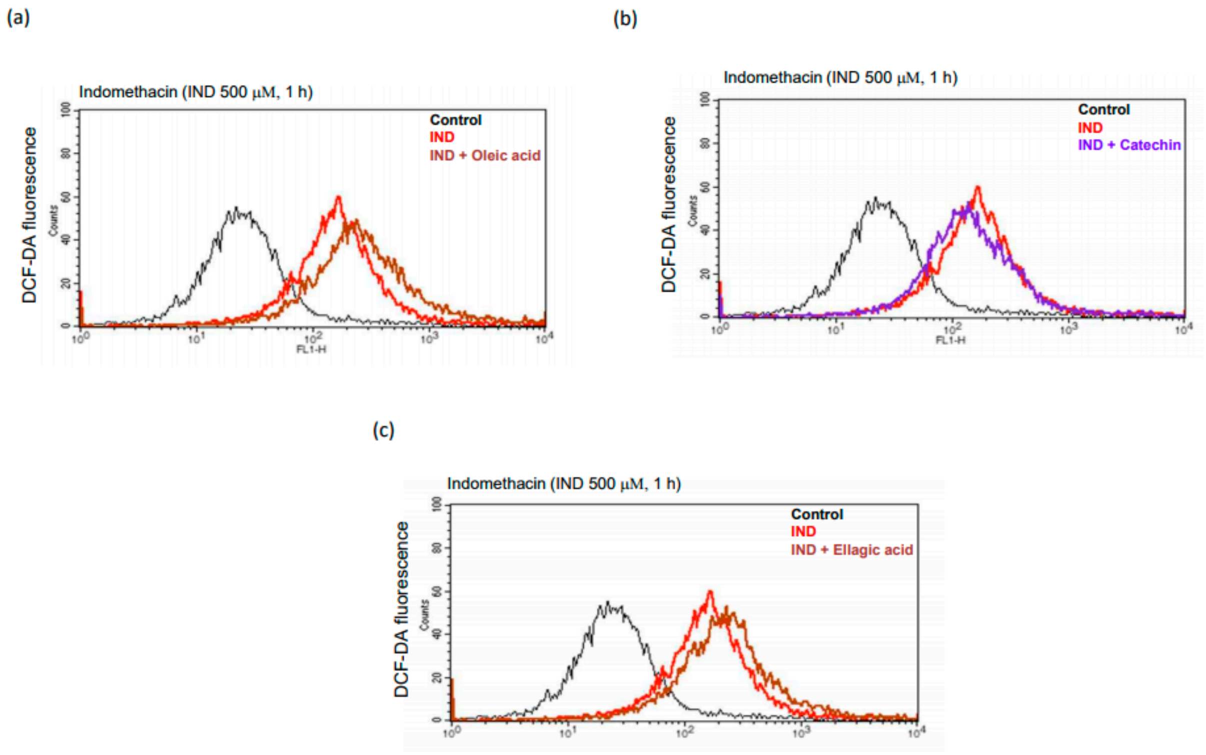

Supplementary Figure S2. DCFDA flow cytometry changes after oleic acid, catechin, and ellagic acid exposure (a) oleic acid for 1hr (b) catechin for 1hr (c) Ellagic acid for 1hr.
